# User vs. software-dependent variability of ELISPOT counts obtained from ten different laboratories

**DOI:** 10.1186/2051-1426-1-S1-P95

**Published:** 2013-11-07

**Authors:** Tameem Ansari, Alexey Karulin, Paul V Lehmann, Srividya Sundararaman

**Affiliations:** 1 R&D, Cellular Technology Ltd., Shaker Hts., OH, USA

## Introduction

In each human donor’s PBMC, there is a defined number of T cells specific for any given antigen. A major goal of immune monitoring with ELISPOT is to measure this number accurately and reproducibly between different laboratories. In ELISPOT assays, cytokine spots produced by antigen-specific T cells show a broad spectrum of sizes and densities over variable background. Therefore, even experienced investigators are likely to come up with different spot counts when subjectively judging the minimal spot size/density to be counted and the maximal spot size for the cut off between single-cell-derived spots vs. those created by cell clusters. This study aims to find out whether statistics-based automated gating can harmonize spot counts obtained in different laboratories.

## Methods

We studied PBMC plated in serial dilutions, with 24 replicates per dilution, to establish the distributional properties of HCMV pp65-induced IFN-γ ELISPOTs. We sent the physical ELISPOT plate and image files obtained from it to ten different laboratories for independent counting. The plate was machine counted by each laboratory relying on either (A) Basic Count, which relies on subjective counting parameters set by the different investigators or (B) SmartCount™, an automated counting method embedded in CTL’s ImmunoSpot® Software that uses statistics-based autogating in conjunction with autothresholding.

## Results

The IFN-γ spots were found to closely follow a Log Normal distribution. When spot counts were established by subjective judgment, Basic Count, the average coefficient of variation (CV) between the mean values for the independent laboratories was 26.7%. Counting with the SmartCount™ method produced counts with an average CV of 6.7% across the laboratories.

## Conclusions

The Log Normal distributional properties of ELISPOTs permits one to automatically set the lower and upper gates for counting spots by means of statistics, achieving a target significance of 95%. Using SmartCount™, which relies on statistics-based autogating in conjunction with autothresholding, spot counts can be harmonized between different investigators and laboratories.

